# Characterization, Evolution, Expression and Functional Divergence of the *DMP* Gene Family in Plants

**DOI:** 10.3390/ijms251910435

**Published:** 2024-09-27

**Authors:** Zeeshan Ahmad, Dingyan Tian, Yan Li, Isah Mansur Aminu, Javaria Tabusam, Yongshan Zhang, Shouhong Zhu

**Affiliations:** 1 National Key Laboratory of Cotton Bio-Breeding and Integrated Utilization, Institute of Cotton Research, Chinese Academy of Agricultural Sciences, Anyang 455000, China; zeeshanpbg@aup.edu.pk (Z.A.); t1109495490@163.com (D.T.);; 2 Western Agricultural Research Center, Chinese Academy of Agricultural Sciences, Changji 831100, China

**Keywords:** *DMP* genes, genome-wide, cotton, characterization, evolution, gene expression

## Abstract

The *DMP* (DOMAIN OF UNKNOWN FUNCTION 679 membrane protein) domain, containing a family of membrane proteins specific to green plants, is involved in numerous biological functions including physiological processes, reproductive development and senescence in *Arabidopsis*, but their evolutionary relationship and biological function in most crops remains unknown. In this study, we scrutinized phylogenetic relationships, gene structure, conserved domains and motifs, promoter regions, gene loss/duplication events and expression patterns. Overall, 240 *DMP*s were identified and analyzed in 24 plant species selected from lower plants to angiosperms. Comprehensive evolutionary analysis revealed that these *DMP*s underwent purifying selection and could be divided into five groups (I–V). *DMP* gene structure showed that it may have undergone an intron loss event during evolution. The five *DMP* groups had the same domains, which were distinct from each other in terms of the number of *DMPs*; group III was the largest, closely followed by group V. The *DMP* promotor region with various *cis*-regulatory elements was predicted to have a potential role in development, hormone induction and abiotic stresses. Based on transcriptomic data, expression profiling revealed that *DMP*s were primarily expressed in reproductive organs and were moderately expressed in other tissues. Evolutionary analysis suggested that gene loss events occurred more frequently than gene duplication events among all groups. Overall, this genome-wide study elucidates the potential function of the *DMP* gene family in selected plant species, but further research is needed in many crops to validate their biological roles.

## 1. Introduction

Membrane proteins are essential for many biological and physiological functions, such as material transport, signal transmission and recognition, cell division and proliferation, and stress responses [1]. For example, *AtHKT1* proteins facilitate the transport of ions and metabolites across cell membranes, which is essential for maintaining cellular homeostasis and overall growth [2] and development by mediating hormone signaling pathways and environmental interactions [3]. Another protein, *AtEC1*, regulates sperm cell recognition with egg cells, and the *AtGEX2* protein plays a vital role in gamete attachment for successful fertilization in *Arabidopsis* [4].

A class of unique membrane proteins present only in green plants are known as *DMP*s (*Domain of unknown function 679 membrane proteins*), a family that typically contains four to five transmembrane domains with cytoplasmic amino and carboxyl termini [5]. In *Arabidopsis thaliana*, a total of ten *DMP*s have been identified and are predicted to have vital roles in various physiological processes, particularly in senescence, reproductive development and various stress responses [6]. Studies have reported that these *DMP*s exhibit different expression patterns in various plant tissues, where *AtDMP1* and *AtDMP2* are involved in developmental senescence and darkness-induced senescence and engage in membrane fission and fusion during senescing and root vacuole biogenesis [7]. Similarly, *AtDMP3* and *AtDMP4* are upregulated in senescent leaves and siliques, demonstrating overlapping roles during senescence. Moreover, *AtDMP4* is co-expressed with developmental programmed cell death genes such as *RIBONUCLEASE3 (RNS3)*, *BIFUNCTIONAL NUCLEASE1 (BFN1)* [8] and *EXITUS1 (EXI1)*, which promote senescence, differentiation-induced and age-induced developmental-programmed cell death [9]. *AtDMP3* and *AtDMP4* are expressed in flowers and roots, while *AtDMP2*, *AtDMP6* and *AtDMP7* are expressed in all plant organs [6]. Moreover, *AtDMP8* and *AtDMP9* are involved in gamete fusion during double fertilization in flowering plants. The expression levels of *DMP*s strongly suggests their involvement in senescence, abscission, vacuole biogenesis, reproductive development and stresses, but some DMP promotor regions do not have the *cis* elements for these activities, which indicates their potential involvement in other cellular activities [5]. 

*DMP*s have been studied, analyzed and characterized in some of the few crop species for their roles and functions. For example, loss of function mutations in *AtDMP*s and mutations in qhir8 of *ZmDMP*s led to the development of an effective haploid induction system in *Arabidopsis thaliana* and *Zea mays*, respectively [10]. Similarly, a haploid induction system was also established in in allotetraploid tobacco [11], polyploid *Brassica napus* [12] and tomato [13] using *DMP* mutants. *AsDMP1* and *AsDMP19* have been found to regulate seed anti-aging and seed longevity in oats (*Avena sativa*) [14], while *Glyma.09G237500* and *Glyma.18G098300* in soybean have been found to control female organ-mediated haploid induction [14]. Furthermore, a systematic study of the *DMP* family in four cotton species identified 58 *DMP*s, and their expression patterns unveiled their probable involvement in vital biological process like reproductive development, plant aging and stress responses [5]. However, few studies have been carried out on the investigation and characterization of the functions of *DMP*s in plant crops. In this regard, we conducted a study on 24 plant species selected from different classes (mosses to angiosperms) in which we identified 240 *DMP* genes. Furthermore, we used bioinformatics tools to comprehensively analyze the physiochemical properties, gene structures, conserved motifs, phylogenetic relationships and promoter regions of the *DMP*s, and have characterized their expression patterns in various crops.

## 2. Results

### 2.1. Identification of the DMP Proteins in Various Species

To identify the *DMP* gene family, BLASTP searches were performed across animal, plant, protist, fungi, and prokaryote protein databases using the ten known DMP amino acid sequences as query sequences from the model plant *Arabidopsis* (Table S7). According to BLAST searches for *DMP*-like proteins, no related amino acid motifs were discovered in mammals, fungi, prokaryotes, or archaea, whereas all higher plants, mosses, and *Chlamydomonas reinhardtii* contain *DMP* homologues. Overall, the BLAST results revealed a total of 1 *DMP* candidate gene in *Chlamydomonas reinhardtii* (Cre), 2 in *Physcomitrella patens* (Pp), 2 in *Selaginella moellendorffii* (Sm), 5 in *Amborella trichopoda* (Atr), 14 in *Aquilegia coerulea* (Aqco), 6 in *Solanum lycopersicum* (Sl), 7 in *Vitis vinifera* (Vv), 10 in *Arabidopsis thaliana* (At), 7 in *Carica papaya* (Cp), 11 in *Gossypium raimondii* (Gr), 8 in *Theobroma cacao* (Tc), 8 in *Citrus sinensis* (Csi), 12 in *Populus trichocarpa* (Ptr), 13 in *Glycine max* (Gm), 13 in *Acorus americanus* (Aa), 12 in *Musa acuminata* (Ma), 7 in *Ananas comosus* (Aco), 11 in *Brachypodium distachyon* (Bd), 19 in *Oryza sativa* (Os), 14 in *Zea mays* (Zm), 15 in *Sorghum bicolor* (Sb), 14 in *Panicum hallii* (Ph), 13 in *Setaria italica* (Si) and 15 in *Setaria viridis* (Sv).

### 2.2. Phylogenetic Analysis of the DMP Gene Family

**Figure 1:**
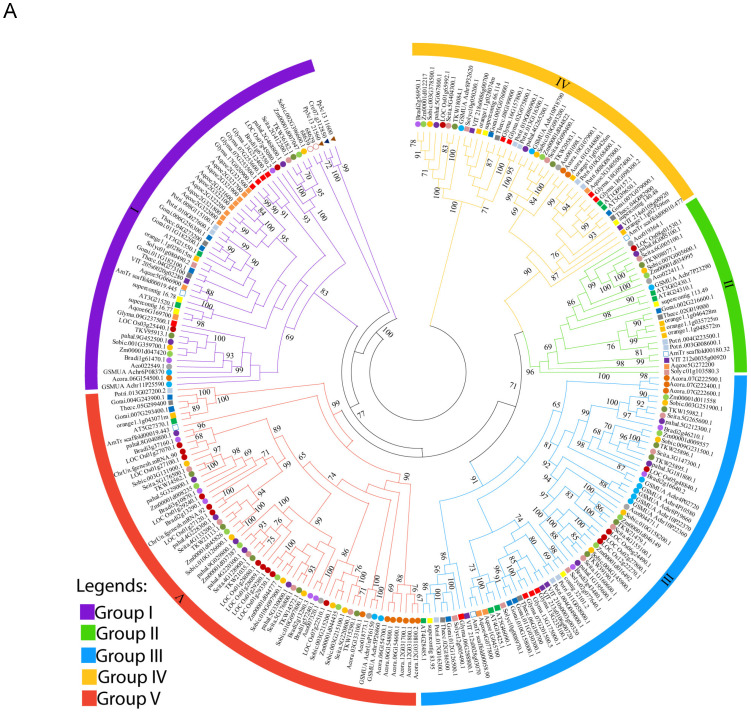

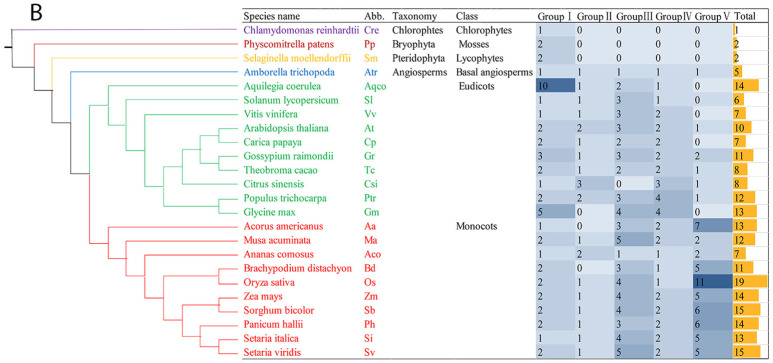
Phylogenetic relationship of 240 *DMP* genes from 24 species, including *Arabidopsis* and cotton, showing a classification of *DMP*s in various groups. (**A**) MEGA 11 software was used to construct phylogenetic trees using the neighbor-joining (NJ) method with 1000 bootstrap replications. Each color shows a different group (I–V). Species are denoted with different colors and symbols in the phylogenetic tree. (**B**) Classification and arrangement of *DMP* genes in various groups based on a phylogenetic tree. Plant species classes are highlighted in various colors on the left. Five groups on the right side are made based on the phylogenetic tree containing *DMP* genes.

To explore the evolutionary history of the *DMP* gene family across plant species, an unrooted phylogenetic tree was constructed in MEGA 11 using the neighbor-joining (NJ) method to analyze 240 orthologous and paralogous *DMP* protein sequences from the 24 species (Figure 1A). The phylogenetic tree of the retrieved protein sequences suggested that the plant *DMP* gene family clustered into five different groups, namely I, II, III, IV and V (Figure 1B). Most *DMP* genes (62) were found in Group III, closely followed by Group V (61), while Group II had the fewest *DMP*s (23). The most primordial green plant, *Chlamydomonas reinhardtii*, a single-celled green alga, has one *DMP* gene. The bryophytes (*P. patens*) and Pteridophyta (*S. moellendorffii*) each have two *DMP* homologue proteins. From a basal angiosperm, *Amborella trichopoda*, the *DMP*s began to diverge into five groups, and each group contained at least a single *DMP* homologue from there on (Figure 1B). The ten eudicot species in the study have several *DMP* homologues, ranging from 6 (*Solanum lycopersicum*) to 14 (*Aquilegia coerulea*), whereas the number of *DMPs* in ten monocot species ranges from seven (*Ananas cosmos*) to 19 (*Oryza sativa*) homologues. Interestingly, in eudicots, the normal frequency of *DMP* gene distribution was one to four, which was similar in most of the groups, except for group I, which had higher number of *DMP* genes in *Aquilegia coerulea* (10)*,* suggesting occurrence of duplication of the genes in this species (Figure 1B). In contrast, no *DMP* was detected in *Solanum lycopersicum*, *Vitis vinefera* and *Glycine max* of group V, suggesting that deletion of the *DMP* genes may have occurred from these species. Likewise in monocots, the normal gene distribution frequency ranged from one to six, which was similar within the groups, except for group V, which had higher number of *DMP* genes in *Oryza sativa* (11), suggesting the occurrence of duplication of the genes in this species. On the other hand, group II of the monocots have normal gene distribution frequency 1, where no *DMP* genes were detected in *Acorus americanus* and *Brachypodiun distachyon,* suggesting loss of *DMP* genes from these species.

Interestingly, in eudicots, the number of *DMP* genes is almost the same in all groups, except group I have higher number of *DMP* genes in *Aquilegia coerulea*, suggesting duplication of *DMP* genes occurred in this species. On the other hand, in some species of group V, we did not find *DMP* genes, indicating deletion of the genes from this group. Likewise in monocots, group V deviates from the normal gene distribution pattern by presenting a higher number of DMPs, providing evidence of gene duplication in the species in this group, whereas no or lower distribution pattern of group II reveals deletion of *DMP* genes from some species in that group.

### 2.3. Gene Structure, Motifs and Domain Analysis of DMPs

**Figure 2:**
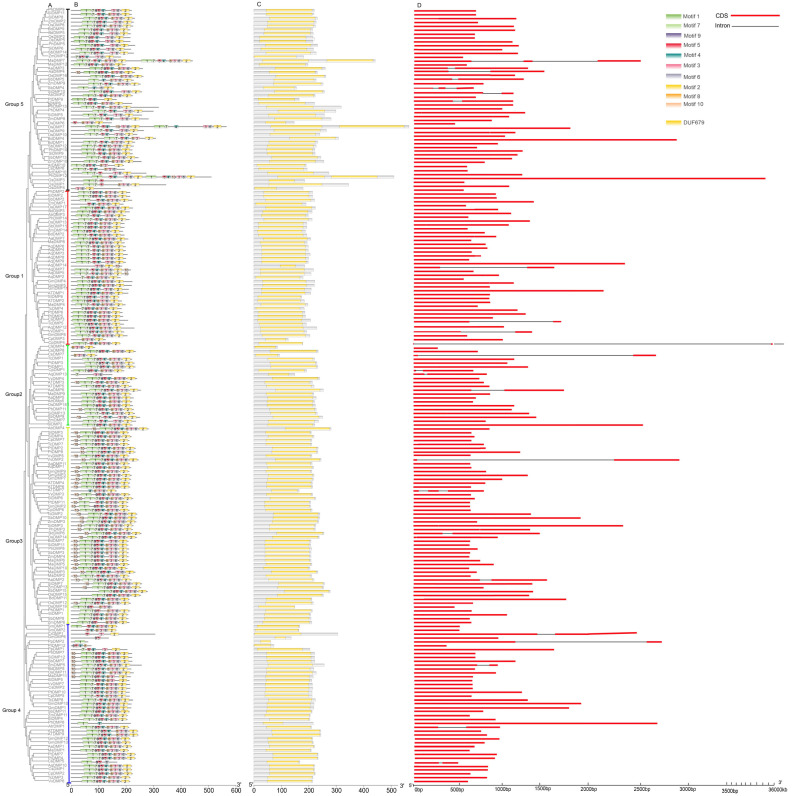
Comparison of the gene structure, conserved motifs and domains in *DMP* genes between *Arabidopsis*, cotton and 22 other species. (**A**) The NJ phylogenetic tree was constructed in MEGA 11 software using *DMP* sequences from 24 species. (**B**) The conserved protein motif composition of all *DMP*s and motif 1–10 is shown in various colored boxes. (**C**) Representation of the domain of *Arabidopsis* and other species. Yellow boxes represent the *DMP* domain in the sequences. (**D**) Exon and Intron structure of *DMP* genes of all Selected species. The red box represents exon or coding region, and the grey line represents intron with the scale at the bottom for measurement length of sequences.

Gene function is related to motifs, domains and structure of the gene. The protein sequences of *DMPs* were used to analyze the gene structure, motifs and conserved domains of all 24 species (Figure 2). Among all species, most of the *DMP*s have no intron structure and have an exon region for a few genes in various groups. Surprisingly, the *DMP* genes have a striking similar intron/exon structure within the same group compared to other groups, i.e., four genes (*CsDMP7*, *CpDMP1*, *AqDmp1*, *AaDMP13*) in Group II harbors two exons and one intron, each of various lengths (Figure 2D). *CsDMP7* exhibits a very large intron region of more than 90% which is unique as compared to other *DMP* genes, suggesting it might have special biological functions. Group III shows the same pattern, with two exons and one intron gene structure observed in *VvDMP2*, *BdDMP6* and *AaDMP2*. Similarly, in Group IV, *ZmDMP5*, *AtrDMP1* and *CsDMP5* each harbor two exons and one intron. In contrast, Group I have *TcDMP5*, *AqDMP14* and *VvDMP1*, each comprising two introns and one exon of highly conserved lengths, in comparison with the gene structure of *CpDMP4*, which contains two very small exons and two very large exons in the same group. Likewise, Group V has a total of seven genes having both exon-/intron-like gene structure. Among them, the gene structure of *MaDMP7* and *SBDMP4* has three exons and two introns of conserved length, while *AaDMP3*, *AaDMP4*, *SbDMP5*, *SiDMP10* and *PtDMP9* bear two exons and one intron. 

The *DMP* sequences were run through MEME Suite (v5.5.7) motif analysis software, and the results revealed that motifs of the *DMP* family were distinct from each other; however, motifs were conserved within the groups (Figure 2B). All the genes were found to have a single *DMP* domain. In total, 10 motifs were identified, whereby each gene had three to ten motifs. Motifs 1 and 2 were commonly present in all groups, and members within groups shared similar motifs, as expected, indicated by the conserved protein architecture within the groups which suggests function similarity. However, functions of these conserved motifs are yet to be elucidated. In contrast, some groups lacking some of the specific motifs such as motif 10 was not present in all members of Groups I and V and most members of Groups II and IV. Motif 9 was missing from most of the members of Groups II and V. However, further research on whether specific motifs have unique functional roles in the plants is needed. These results fully support our phylogenic analysis, and such diversity in motif composition within subfamilies might be explained by their functional diversification.

The protein sequences of the 24 selected species from different families were subjected to domain analysis to better understand the divergence among five groups of *DMP*s. Results of logo sequences revealed that all sequences of *DMP*’s share only one typical conserved *DUF679/DMP* domain (Figure 2C) and having two to five trans-membrane domains like *Arabidopsis* (Table S6; Figures S2–S6). All trans-membrane domains were within the *DUF/DMP* domain, and the *DMP* domain locus within the same group was highly conserved, i.e., the *DMP* proteins in Groups I and II are most alike (Figure 2C, Figures S2 and S3). These results suggest that all *DMP* sequences among the species with the same *DUF/DMP* domain may have the same functions in all the species. However, identification of the specific functions of these genes requires further research.

### 2.4. Promoter Region Analysis in Dicots and Monocots

**Figure 3:**
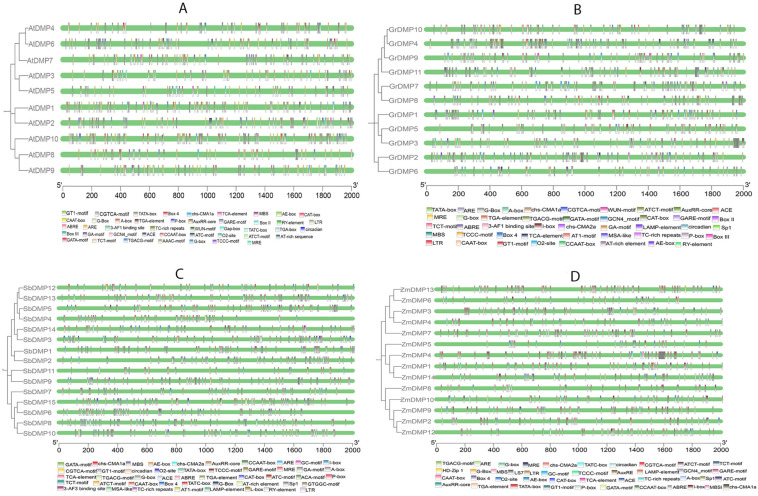
Promoter region analysis of *DMP* sequences from two monocot and two dicot species along with phylogenetic tree of the *DMP*s for each species separately. (**A**,**B**) = Dicots (*Arabidopsis thaliana* and *Gossypium raimondi*). (**C**,**D**) = Monocots (*Sorghum bicolor* and *Zea mays*).

*Cis* elements in the upstream promoter sequence often influence gene expression. *Cis* elements in the non-coding DNA upstream of the gene transcription start site control gene expression in various settings and may be responsive to stress receptors or differentially regulated in tissues. Analyzing *cis* elements involved in *DMP* gene regulation can assist in understanding the gene’s regulatory mechanism and its probable functions. To identify the *Cis*-acting elements in the promoter region of *DMP*s, a 5′ terminal 2000 bp upstream region of the start codon was selected and analyzed using the PLANTCARE database [15]. Results disclosed that the promoter regions of *DMP*s of both monocot (*Sorghum bicolor* and *Zea mays*) and dicot (*Arabidopsis thaliana* and *Gossypium raimondii*) species had similar *cis*-acting elements, including responsive elements, and development, phytohormones and stress response related elements from 59 *DMP* genes (Figure 3).

Among the *cis* elements involved in growth and development of plants, the light-responsive elements accounted for more than 50% and were distributed along the promoter regions in both monocots and dicots (Table S3). These elements include Sp1, L-box, LS7, AE-box, I-box, G-box, Gap box, Box II, Box IV, GA motif, ATC motif, AAAC motif, GTI-motif and TCT motif, which are present in the promoter regions of *AtDMP5*, *GrDMP4*, *GrDMP7*, *GrDMP9*, *ATDMP1*, *AtDMP2* and *AtDMP3* (Table S8a). *Cis* elements responsible for metabolism regulation, plant growth and development include circadian, O_2_ cite and HD-Zip1 (present in the promoter region of *AtDMp8*, *AtDMP9*, *AtDMP10*, *GrDMP1, GrDMP9*, *ZmDMP6*, *ZmDMP7*, *SbDMP1* and *SbDMP8*), meristem expression-related CAT box (in the promoter region of *AtDMP2*, *AtDMP9*, *GrDmp5*, *GrDMP6*, *GrDMP8*, *ZmDMP3*, *ZmDMP4*, *SbDMP3* and *SbDMP7*), endospermic expression-related GCN4 (in the promoter region of *ATDMP4*, *GrDMP9* and *ZmDMP9*), and seed-specific regulation-related RY elements (in the promoter region of *ATDMP10*, *GrDMP3* and *SbDMP10*) (Table S8a–c). The presence of GCN4 and RY-elements in the above genes suggests that DMPs may have vital roles in plant reproductive development.

Phytohormone-related elements, including TGA box, TGA element and AuxRR-core (in promoters of *AtDMP1*, *GrDMP10*, *SbDMP2* and *ZmDMP3*) involved in auxin responsiveness, TCA element responsible for salicylic acid responsiveness (Table S3), GARE-motif and TATC-box (in promoters of *AtDMP7*, *GrDMP5*, *SbDMP7* and *ZmDMP10*) involved in gibberellin responsiveness, and CGTCA- and TGACG-motifs involved in methyl jasmonic acid responsiveness signaling, were identified in the promoter regions of *DMP*s (Table S8a–d). Most of the *cis*-acting elements such as ABRE and AAGAA that are responsible for abscisic acid responsiveness were present in promoter regions of *AtDMP5*, *AtDMP8*, *GrDMP1* and *GrDMP2*. However, most important cytokinin related *cis* elements were not found in any of the promoter regions.

In the promoter region of *DMP*s, we also identified *cis* elements related to stress responses; in particular, elements related to drought stress were enriched, such as dehydration-related MYC, Myb, Myc and Myb binding cites (*GrDMP2*, *SbDMP15* and *ZmDMP9*), MBS involved in drought inducibility, TC-rich repeats for defense and stress responsiveness, and drought, salt and low temperature stress-responsive DRE cores (Figure 3). *Cis*-acting elements such as LTR and STRE (in promoter regions of *AtDMP4*, *AtDMP8*, *ZmDMP1*, *ZmDMP2*, *SbDMP2*, *SbDMP3*) that are involved in low temperature responsiveness were also identified (Table S3). Other *cis* elements like ARE (in promoter regions of *AtDMP6*, *GrDMP11*, *SbDMP14* and *ZmDMP2*) for anaerobic induction, WUN-motif, WRE3 and W-box (in promoters of *AtDMP9* and *GrDMP6*) involved in wound responsiveness were not present in monocot species (Table S8c,d).

### 2.5. Duplication and Loss of DMP Genes in Plants during Evolution

**Figure 4:**
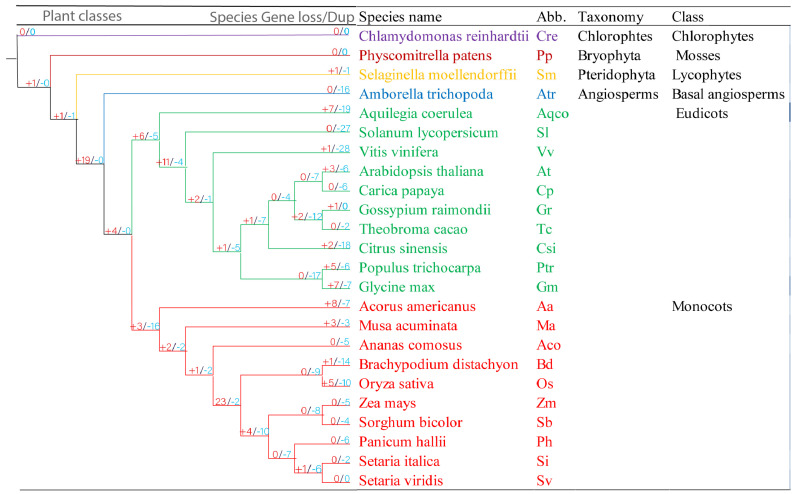
Evolutionary events in the *DMP* gene family in plants. Numbers of gene losses are shown in red color after “+” and duplications in blue color after “−”in the phylogenetic tree on the left side.

We performed gene duplication and loss analysis in plants using NOTUNG (v2.9.1.5) software to better understand the evolutionary events that occurred among these *DMP* genes [16]. Getting *DMP* genes from phylogenetic analyses at various phases of evolution, we further determined if internal nodes within each group were related to gene duplication, gene loss or lineage divergence events (Figure 4).

In *Chlamydomonas reinhardtii*, no gene duplication and gene loss event were detected. Evolving from chlorophytes to mosses, one gene duplication event occurred; however, no gene loss event was found. During the emergence of lycophytes, one gene duplication and one gene loss event took place. *Selaginella moellendorffii* in this taxonomic class also experienced one gene duplication and gene loss event. However, in the lower angiosperms, 19 genes were duplicated, and no gene was lost in the event. From *Amborella trichopoda*, being a lower angiosperm, the *DMP*s began to diverge into five groups, and each group contained at least a single *DMP* homologue within the group, showing more evolution, gene duplication and gene loss events (Figure S1). However, this important species lost 16 *DMP* genes during the evolution process. In eudicots, including ten important species, four gene duplication events occurred while no gene was lost. *Arabidopsis*, an important model species of this class, went through three gene duplication and six gene lost events during the evolution process. In monocots, containing ten species, three genes were duplicated while 16 genes were lost from this class. *Oryza sativa*, an important model species in the group, experienced five gene duplications and ten gene losses. However, *Setaria virdis* displayed no gene duplication or gene loss event. 

### 2.6. Tissue Expression Levels of DMPs in Monocots and Eudicots

**Figure 5:**
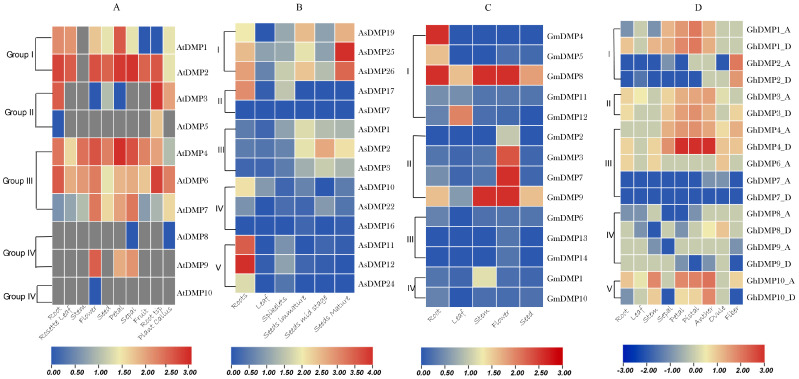
Expression profiles of *DMP* genes in various organ tissue from two monocot and two dicot species. Raw RNA-seq data were procured from relevant databases. (**A**) *Arabidopsis thaliana* from TAIR database (TAIR, v.11, http://www.arabidopsis.org). (**B**) *Avena sativa* and (**C**) *Glycine max* from Phytozome (https://phytozome.jgi.doe.gov/pz/portal.html/, accessed on 5 March 2024). (**D**) *Gossypium hirsutum* from COTTONGEN (http://www.cottongen.org/, accessed on 7 March 2024) and the Cotton Functional Genomics Database (CottonFGD, https://cottonfgd.org/)*. DMP* gene expression levels are represented with various colors, with blue color indicating the least expression and red indicates the highest expression. Eudicot species (**A**,**C**,**D**); monocot species (**B**).

We performed the expression analysis of *DMP*s in various tissues across two monocot and two eudicot species using the transcriptomic data from various databases (Table S4). The results showed that different *DMP*s expressed in most of the organ tissues of plants, particularly in the reproductive organs as well as various growth and developmental stages of plants, suggests that these genes may have different functions and roles in various crop species (Figure 5). 

*DMP* genes of Group I were highly expressed in roots, leaves, flowers and petals in monocot species, while in eudicots, expression of *DMP*s was limited to just roots and seeds. In Group II, the monocot species (*Avena sativa*) *AsDMP*s such as *AsDMP12*, *AsDMP11*, *AsDMP17* and *AsDMP26* are greatly expressed just in roots (Figure 5B). However, eudicots followed a different pattern, where *AtDMP*s (*Arabidopsis thaliana*) such as *AtDMP1* and *AtDMP2* are highly expressed in roots, *GmDMP*s (*Glycine max*) such as *GmDMP8* and *GmDMP5* are highly expressed in flowers, and *GrDM*s (*Gossypium raimondii*) are highly expressed in petals, pistils and anthers (Figure 5A,C,D). The Group III monocot *AsDMP1*, *AsDMP2* and *AsDMP3* were rarely expressed in roots, whereas in dicots, *AtDMP4* and *AtDMP6* of *Arabidopsis* and *GrDMP4* and *GrDMP6* of cotton were highly expressed in flowers and reproductive organs like sepals, petals and seeds. However, *GmDMP*s were not expressed in any of the tissues (Figure 5C). In Group IV, the monocot *AsDMP10* and *AsDMP22* were highly expressed in roots and leaves. In contrast, in dicot species, *AtDMP9*, *GmDMP1* and *GrDMP9A* were greatly expressed in flower, sepal and petal tissues. The other gene in this group was expressed in various tissues. For Group V, *AsDMP11*, *AsDMP12* and *AsDMP24* of the monocot species *Avena sativa* were predominantly expressed in roots. However, dicot *DMP*s like *AtDMP10*, *GrDMP10A* and *GrDMP10B* were expressed in the reproductive tissues as well as in vegetative tissues of the plants.

### 2.7. Tissues Expression of DMPs in Cotton

**Figure 6:**
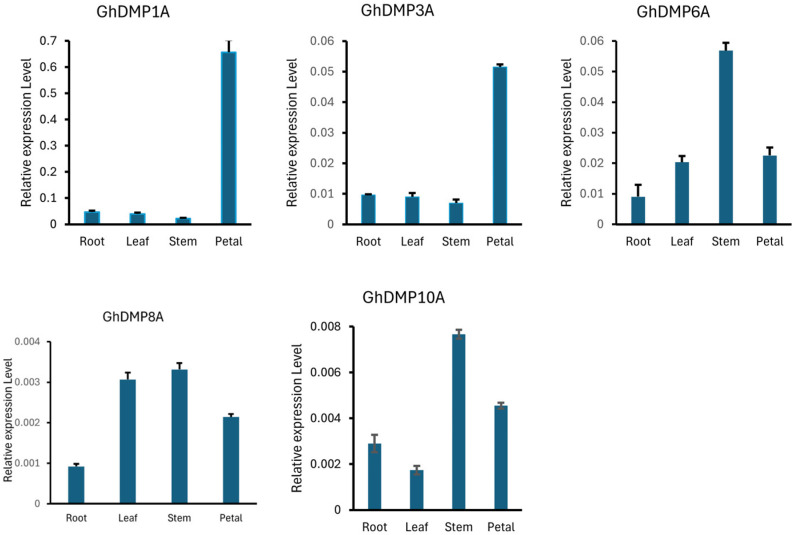
Expression levels of *DMP* sequences in various tissues of cotton. *GhActin* was used as a reference. Error bars denote standard deviation measured from three experiments.

We selected five *DMP* genes (*GhDMP1A*, *GhDMP3A*, *GhDMP6A*, *GhDMP8A* and *GhDMP10A*) based on RNA-Seq data to confirm the expression profiles of *DMP* genes in various tissues of *Gossypium hirsutum* by qRT-PCR. The gene expression patterns from qRT-PCR analysis showed a similar trend to the pattern detected using the RNA-seq data, as shown in Figure 6. *GhDMP1A* and *GhDMP3A* dominantly expressed in petals with the least expression in vegetative parts, whereas *GhDMP6A*, *GhDMP8A* and *GhDMP10A* expressed in reproductive as well as vegetative tissues of the plant (Table S5). Based on these results, *DMP* genes may have been involved in reproductive functions rather than playing a role in vegetative growth and development of plants.

## 3. Discussion

The *DMP* sequence resembled known membrane proteins, transporters or channels in any kingdom [5], suggesting functions in processes that also occur in plants. These protein sequences are limited to green plants solely, evolved and expanded particularly in flowering plant lineages [5,17]. In this study, we used various bioinformatics tools and publicly available transcriptomic datasets to mine genomes of different crops for *DUF679* family genes. Various numbers of *DMP* genes were identified in different crop species from distinct plant classes. Polyploid and complex genomes, and gene duplication and loss events during the evolutionary process in plants may increase or decrease the number of *DMPs.* To construct a phylogenetic tree, we analyzed *Arabidopsis DMP* sequences along with 23 other species from various classes of plants and found that all species’ *DMP* members could be clustered into five groups, the same as in *Arabidopsis* (Figure 1A). Groupwise, the gene frequency distribution pattern was the same within groups, with some exceptions; for example, in dicot Group I, all members had almost equal numbers of genes except *Aquilegia coerulea*, which had eight times more genes than other species. Likewise, in monocot Group V, the number of genes in *Oryza sativa* was double that of other species in the group, which might be due to duplication of genes in their larger genome over time in comparison to smaller genomes (Figure 1B). Structure analysis of *DMP* sequences revealed that most protein sequences in all species had no introns, with some exceptions having single or double intron/exon structure (Figure 2D), indicating potential special functions of the single or double intron/exon genes in various plants. These results support previous studies on *DMP*s conducted in *Arabidopsis* and cotton [6,7]. Similar to our studies, previous findings on genetic sequencing also suggested that initially, eukaryotic predecessors had intron-rich genes individually, and most of them experienced losses of introns during the process of evolution [18]. Motif analysis results disclosed certain differences in conserved motifs, suggesting that *DMP*s take part in various biological functions of growth and development. Three motifs, namely, motif1, motif2 and motif3, were conserved in all sequences of *DMP*s particularly with motif1 and motif2 of the DUF679 domain among the species, while other were restricted to specific genes (Figure 2C). Moreover, the motif pattern was the same within the same group while various groups showed different combination of motifs. However, most of the amino acids of *DMP*s contain a single *DMP* domain with four to five transmembrane domains (Table S6; Figures S2–S6), which indicates that *DMP*s have a common ancestor and evolved independently and may have the same special functions. Some earlier studies proposed that a single amino acid mutation in the initial transmembrane of *DMP* gene in *qhir8* in maize can induce haploids [19,20]. Furthermore, based on transcriptomic data, the expression pattern of *DMP* genes among four monocot and dicot species was almost similar (Table S4). Most of the *DMP* genes widely expressed in reproductive organs, some in vegetative parts, showing that *DMPs* may play a reproductive role rather than playing a part in vegetative growth in general [5,14]. Consistent with this, the qRT PCR analysis showed that each representative *DMP* gene from the group was expressed widely in floral organs and to some extent in vegetative tissues (Figure 6). These results signify previous findings that *DMP* may have roles in fertility and reproductivity [5]. Looking briefly at the functions of *DMP*s, *DMP4* promotes senescence and interacts with *DMP1* [21], *AtDMP2* and *AtDMP3* involved in programmed cell death [14,22]. Also, other studies reported that mutation in *DMP8* and *DMP9* in *Arabidopsis* resulted in decreased fertility and seed setting rates [22,23]. Equally, a recent study in soybean also demonstrated the probable involvement of *DMP8* and *DMP9* in pollination and fertilization. Previously, it was determined that *DMP1* is involved in membrane fission and fusion during leaf senescence and vacuole biogenesis in roots [6,24]. Additionally, research in oat has found the involvement of *DMP1* in anti-aging and seed longevity [25]. It can be concluded that *DMP*s play a vital role in development and fertility of monocot and dicot species. However, the functions of all the *DMP* genes are still unknown in many crop species, and further research is needed to investigate these thoroughly.

Looking into gene loss and duplication events, primordial plants contain a maximum of two *DMP*s per species, with no gene loss or duplication events recorded. *DMP* genes began to diverge in the basal angiosperms by having at least five copies of *DMP*s in a species (Figure 4). Thereafter, the greatest number of genes was duplicated in monocot species, while high numbers of *DMP* genes were frequently lost from dicot species, signifying that rapid birth and death events occurred in *DMP*s during that time (Figure S1). A single copy of genes in any genome is enough for essential viability, and loss or duplications leads to change of functions. For example, *Cter-SUN* proteins in *Caenorhabditis elegans* and *Schizosaccharomyces pombe* were responsible for nuclear migration and duplication of SBPs (spindle pole bodies) [26,27] However, when *Cter-SUN* proteins duplicated into *SUN1* and *SUN2*, they started to have vital roles in chromosome movement [28]. These results suggest that *DMP* functions may have changed over time due to frequent loss or duplication events. 

We studied the distribution and frequency of *cis*-acting regulatory elements (CAREs) in four crop species (monocots and dicots) to explain the possible *cis*-regulatory roles of *DMP*s in growth and stress responses. Interestingly, all the studied species from monocots and dicots had almost the same *cis* elements and promoter regions with minor exceptions (Figure 3; Table S8a–d). Regarding growth, light responsive elements (LREs) were found to be predominant in *DMP* promoter regions. G-Box, Box4, GTI-motif, LS7 and I-box were the most common *cis* elements and are considered critical for regulating light-mediated transcriptional activity [29,30]. The most common G-box (CACGTG) is reported to be involved in regulation of chlorophyll biosynthesis in *Arabidopsis* [31], while the GATA motif has a vital role in tissue specificity and development of tissues [32]. G-box and I-box present in the promoter regions of *DMP*s are considered to be photosynthetic-responsive elements, and combined, both are essential for activation of phytochrome, cryptochrome and plastid signals [33,34]. Interestingly, *Arabidopsis*, maize, oat and *G. raimondii* had a site for an sp1 (GGGCGG) element (Table S8a–d), a mammalian promoter element associated with the regulation of a large number of housekeeping and tissue-specific genes [35]. Previous studies concluded that it was absent from the rice genome, lacking significant functions [36]. Relating growth and development of the plant O_2_-site, GCN4_motif and activation sequence 1 (TGACG) were frequently found in all of the *DMP* sequences (Table S3; Table S8a,c). Activation sequence 1 element has been found to play a role in auxin- or salicylic acid-dependent enhanced expression in leaves [37]. Furthermore, we identified seed-specific regulation related to the RY element in promoter regions of numerous *DMP*s (Figure 3; Table S8a–d). This element interacts and binds with *ABI3* protein and *FUS3* protein during development of mature seeds in *Arabidopsis* [38].

Phytohormones, i.e., plant hormones, are chemical substances that regulate diverse biological processes in plants, involving growth, development and responses to environmental stresses [39]. Key phytohormones controlling regulatory elements including methyl jasmonic acid responsive elements, abscisic acid responsive, auxins and gibberellin responsive elements were present in promoters of the studied *DMP*s. Among them, (abscisic acid responsive elements (ABREs) have higher binding sites in most of the *DMP* promoter regions (Figure 3; Table S8a–d), acting as regulators of biological processes like stomatal closure, seed and bud dormancy, and plant responses to drought, cold, heat and saline stresses [40]. Methyl jasmonic acid responsive elements (CGTCA-motif and TGACG-motif) were found in several promoter regions (Table S3). They are considered vital in the regulation of *TF-mediated* gene [41]. For instance, the TGACG motif from the rice promoter region regulates *12-oxophytodienoic acid reductase-1* and plays a crucial role in defense responses [42]. Moreover, some promoter regions have auxin responsive elements (Figure 3; Table S3), which induces the expression of *WOX11* and its homologue *WOX12* gene, thus stimulating the cell fate transition [43].

*DMP* promoter regions also contained *cis*-regulatory elements accountable for modulating gene expression in response to various stresses. For stresses, DRE cores, MBS, MYC, MBSI, Myb, Myc and Myb-binding sites were identified in most promoter regions (Figure 3; Table S3). Previous reports suggested they play inevitable roles in drought-inducible expression, indicating that *DMP* expression is also related to abiotic stress [44]. Additionally, the presence of low temperature-responsive (LTR) elements in some of the *DMP* sequence promoters shows its involvement in cold stress [45]. Some of the sequences contained promoter regions for anaerobic-responsive elements (AREs), which are known for having a role in response to low oxygen and dehydration [46]. The WUN-motif and W-box (TTGACC) were present in promoter regions of *AtDMP9* and *GrDMP6* (Figure 3; Table S8c,d), and is believed to help plants in healing during stress conditions [47]. Furthermore, the W-box element interacts with *WRKY* transcription factors to regulate the expression of defense related genes and helps plant seeds during dormancy and senescence [48]. The presence of diverse stress-responsive *cis*-regulator elements and varied expression patterns of *DMP*s using public data indicates their potential roles in improving stress tolerance and survival (Figure 3). However, this research only preliminarily analyzed phylogenetic relations and characterized basic functions of *DMP*s, and further research is needed on the functional validation of genes to understand their relevance during stress and various biological processes in various monocot and dicot crop species.

## 4. Materials and Methods

### 4.1. Identification and Retrieval of DMP Gene Family

The published protein sequences of *Arabidopsis thaliana* (*AtDMP*s) were retrieved from the Arabidopsis Information Resource database (TAIR, v.11, http://www.arabidopsis.org/, accessed on 2 March 2024), while the genome sequences of *Chlamydomonas reinhardtii* (v5.6), *Physcomitrella patens* (v3.3), *Selaginella moellendorffii* (v1.0), *Amborella trichopoda* (v1.0), *Aquilegia coerulea* (v3.1), *Solanum lycopersicum* (ITAG4.0), *Vitis vinifera* (v2.1), *Carica papaya* (v0.4), *Theobroma cacao* (v2.1), *Citrus sinensis* (v1.1), *Populus trichorpa* (v3.1), *Glycine max* (Wm82.a4.v1), *Acorus americanus* (v1.1), *Musa acuminata* (v1.0), *Ananas comosus* v3.0, *Brachypodium distachyon* v3.1, *Oryza sativa* (v7.0), *Zea mays* (RefGen_V4), *Sorghum bicolor* (v3.1.1), *Panicum hallii* (v3.1), *Setaria italica* (v2.2) and *Setaria viridis* (v2.0) were downloaded from phytozome (https://phytozome.jgi.doe.gov/pz/portal.html/, accessed on 5 March 2024). The genome sequences of *Gossypium raimondii* (CRIv2.1) were acquired from the COTTONGEN (http://www.cottongen.org/, accessed on 7 March 2024) [49] and reconfirmed in the Cotton Functional Genomics Database (CottonFGD) (https://cottonfgd.org/, accessed on 11 March 2024) [50].

*Arabidopsis* protein sequences were used as query sequences (Table S7), and the BLASTP program was used to search the candidate *DMP* sequences in the above species protein databases. Subsequently, the acquired protein sequences were verified for the *DMP* domain (IPR007770) in the candidate sequences using SMART (http://smart.embl.de/, accessed on 18 March 2024) [51] and Interproscan 05 (https://www.ebi.ac.uk/jdispatcher/pfa/iprscan5, accessed on 22 March 2024) [52], and eventually the DMP sequences were identified. Later, Deep TMHMM server v.2.0 (https://services.healthtech.dtu.dk/services/TMHMM-2.0/, accessed on 25 March 2024) was used to predict the number of transmembrane domains in the targeted sequences [53]. This information, along with gene numbers, is presented in Supplementary Table S1.

### 4.2. Amino-Acid Sequence Alignment and Phylogenetic Analysis

The full-length protein sequences of *DMP*s were multiple aligned using DNAMan v2.0 for multiple sequence logo analysis, the transmembrane domain sequences of *DMP*s from all the selected species were aligned, and the multiple alignment result was used for generating sequence logos through an online tool, WEBLOGO (https://weblogo.berkeley.edu/logo.cgi, accessed on 27 March 2024) [54]. The extracted *DMP* sequences from 24 plant species were aligned using MEGA Suite software (v.11.0) followed by a neighbor-joining (NJ) tree method to construct a phylogenetic tree [55]. The phylogenetic analysis was performed using bootstrap method with 1000 replicates and the substitution was then evaluated by a Poisson model with default parameters.

### 4.3. Conserved Motifs, Domain and Gene Structure Analysis

The conserved motifs of *DMP* sequences were predicted using Multiple Expectation Maximization for Motif Elicitation (MEME suite v.5.5.5) online website (https://meme-suite.org/meme/info/status?service=MEME&id=appMEME_5.5.51716255002353-1793066522, accessed on 29 May 2024) for motif identification [56]. For motif identification, the *p*-value was kept lower than 10^−5^, mode was set to “classic mode”, the site distributions were “zero or once per sequence”, the number of motifs was set to “10”, and the width of motifs was set to “between 6 and 50”. The *DMP* gene exon/intron structures, conserved motifs and domains along with phylogenetic tree was drawn and visualized with TBtools software (v.2.102) [57], and the Gene Structure Display Server (GSDS v2.0, https://gsds.gao-lab.org/index.php, accessed on 9 June 2024) by using NWK file from the phylogenetic tree, MAST file from the MEME website, CDS and genome files of all selected species [17]. 

### 4.4. Promoter Analysis of Monocot and Eudicot Species

Among the studied species, two monocot (*S. bicolor* and *Z. mays*) and two dicot (*A. thaliana* and *G. raimondii*) species were selected, and their promoter regions were analyzed to predict the *cis*-acting elements and compared with each other to find any differences among them. The DNA sequences of the 2000 bp upstream region of the start codon for *DMP*s were retrieved from Phytozome v.13 (https://phytozome-next.jgi.doe.gov/, accessed on 7 April 2024) website for selected species. The obtained sequences were then submitted to the PlantCARE website (https://bioinformatics.psb.ugent.be/webtools/plantcare/html/, accessed on 17 June 2024) to predict and analyze the *cis*-regulator elements related to phytohormones, plant growth and development, stresses and light responses in the promoter region of *DMP*s [15]. TBtool (v2.119) software was used to draw and visualize the graphics for *cis*-acting elements.

### 4.5. Gene Loss and Duplication Event Analysis

The plant species tree was constructed using an online website (Time Tree v.5.0, https://www.timetree.org/, accessed on 3 April 2024) [58] in NWK format, while the gene tree was adapted from MEGA (v.11) software [59] in NWK file format using protein sequences. Sequences were first aligned in MEGA and then a NWK tree was constructed for the protein sequences. Similarly, a separate NWK species tree was constructed for all species from which the sequences were extracted. The gene tree was then reconciled with the plant species tree by Notung-DM software (v.2.9.1.5) with default parameters to obtain the trees’ gene loss and duplication events [16].

### 4.6. Expression Profiles of DMPs in Monocots and Eudicots

RNA-seq data were downloaded from the TAIR database (v.11, http://www.arabidopsis.org, 10 May 2024) for *Arabidopsis thaliana*, from the Cotton Omics database (http://cotton.zju.edu.cn/10.rnasearch.html, accessed on 13 May 2024) for cotton [60] and from the Ensemble Plants (v.59) database (https://plants.ensembl.org/, accessed on 16 May 2024) for *Avena sativa* and *Glycine max*. Individual experimental design and sampling methods used for RNA extraction in different monocot and dicot species are explained below: 

For *Arabidopsis*, total RNA was isolated from various tissues such as young leaves (20, 34 and 50 days after sowing), green and ageing cauline leaves, siliques, root tips, plant callus, sepals, petals, young and old stems. For young stems, the upper main part was used, whereas old stems included only the brown part below the first node. Roots and root tips samples were collected from lower parts of plants covered in soil [6].

Oat seeds of the Baiyan 2 variety were utilized in the experiment. They were naturally stored for 0, 1, 2 and 3 years at an average temperature of 10 °C. The harvested seeds were artificially aged by lowering their moisture content to 10–14% and storing them for 24, 48, 72 and 96 h at 45 °C and 95% humidity. Afterwards, the seeds were dried and kept at 4 °C. Unaged seeds were used as control treatment [25].

Soybean seeds of the Williams 82 variety were cultivated at 25 °C with 16 h of light and 8 h of darkness. After 60 days, RNA was collected from leaves, stems and flowers. In total, three fresh leaves were cut off from the top, and stems were cut 3 cm below the top part of the plant. Plant floral parts such as petals, sepals, pistils and pollen were taken from 100 open flowers on a total of four plants [14].

The cotton (*Gossipium hirsutum*) cultivar Zhongmiansuo 100 was used to extract total RNA from stems, roots, sepals, leaves, petals, bracts, stigma, anthers (flower buds of different sizes: <3 mm, 3–5 mm, 5–8 mm and >8 mm), pollen and fiber (6, 12, 18 and 24 days post anthesis). The RN38-EASYspin-Plus Plant RNA kit was used for RNA extraction [5].

Different isolation kits were used for DNA extraction in the above experiments, followed by agarose gel electrophoresis to assess the quality and Nanodrop or Nanopro spectrophotometry to determine the concentration and purity of the samples. Thereafter, RNA was reverse transcribed with the prime Script^TM^ RT reagent kit and gDNA Eraser. Specific primers were used in every experiment according to their materials. PCR conditions were consistent across all tests, beginning with denaturation at 95 °C for 15 min, followed by amplification (40 cycles) at 95 °C for 10 s and annealing at 58 °C for 30 s. The melt curve analysis was set to default, which includes 1 s at 95 °C, 15 s at 65 °C, and 1 s at 95 °C. All experiments were replicated thrice, and the 2^−ΔΔCt^ protocol was applied to obtain the expression levels of genes. Heat map charts along with phylogenetic trees were then generated through TBtool (v2.119) software according to gene expression values (fragments per kilobase million, FPKM). 

### 4.7. RNA Extraction and qRT-PCR Analysis

Total RNA was extracted from various tissues of the cotton (*Gossypium hirsutum*) cultivar, including root, leaf, stem, and petal, using an RN38-EASYspin-Plus Plant RNA Kit (Aid lab Co., Ltd., Beijing, China). A 60-day-old plant was selected to take samples of newly formed leaves from the top and a stem sample was taken 3 cm below the shoot apex. Five newly opened flowers were selected from each plant (with a total of 10 plants), and fresh roots along with tips were sampled. Samples were taken from the cotton cultivars and immediately stored in liquid nitrogen for subsequent RNA extraction. The pure RNA was then reverse transcribed to cDNA using a Prime Script™ RT reagent kit (Takara Biomedical Technology Co., Ltd., Beijing, China) following the manufacturer’s protocols. Bio-Rad 7500 fast fluorescence quantitative PCR system with SYBR^®^ Premix Ex *Taq*™ (Tli RNaseH Plus, Takara Biomedical Technology Co., Ltd., Beijing, China) was used for qRT-PCR amplifications according to the manufacturer’s instructions. The PCR machine was set for primary denaturation at 95 °C for 30 s followed by 40 cycles of amplifications at 95 °C for 5 s and at 65 °C for 30 s. The The qRT-PCR experiments compromised biological replicates and three technical replicates with the gene *GhActin* serving as the internal reference, whereas the relative gene expression levels of *GhDMP*s were calculate by the 2^−ΔΔCt^ method [61]. As *DMP* genes are divided into five groups, an individual representative gene was selected from each group and then their expression levels were compared. The specific primers of selected genes for qRT-PCR are listed in Supplementary Table S2.

## 5. Conclusions

This study provides a comprehensive genome-wide analysis of the *DMP* gene family across 24 plant species, highlighting its evolutionary history, gene structure and potential biological functions. Our findings suggest that *DMP* genes have undergone purifying selection and significant evolutionary changes, including intron loss and gene loss events, contributing to their current diversity. The conserved domain structure across five distinct groups indicates functional conservation, while promoter analysis and expression profiling suggest a key role for *DMP* genes in reproductive development, hormone response and abiotic stress adaptation. Despite these insights, further functional studies are required, particularly in major crop species, to fully understand the biological roles of *DMP* genes.

## Data Availability

Data are contained within the article or Supplementary Materials.

## Supplementary Materials

The following supporting information can be downloaded at: https://www.mdpi.com/article/10.3390/ijms251910435/s1.
