# Supplementary figures and images for: Characterization, Evolution, Expression and Functional Divergence of the DMP Gene Family in Plants
Source: Int J Mol Sci. 2024 Sep 27;25(19):10435. doi: 10.3390/ijms251910435 (PMC11477165; doi:10.3390/ijms251910435)

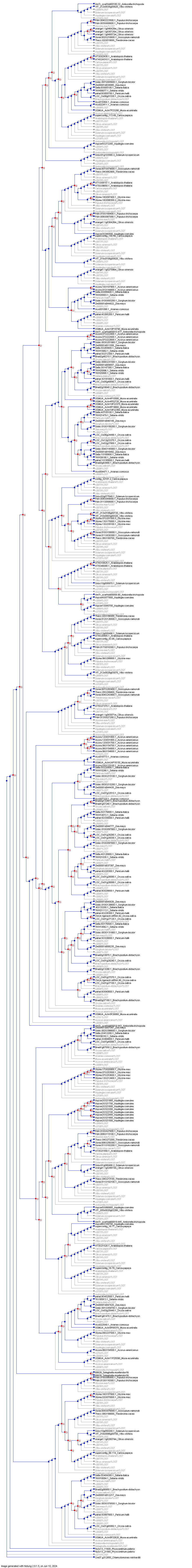

Supplement: Supplementary file 1 [file ijms-25-10435-s001.zip › Figure S1.png]

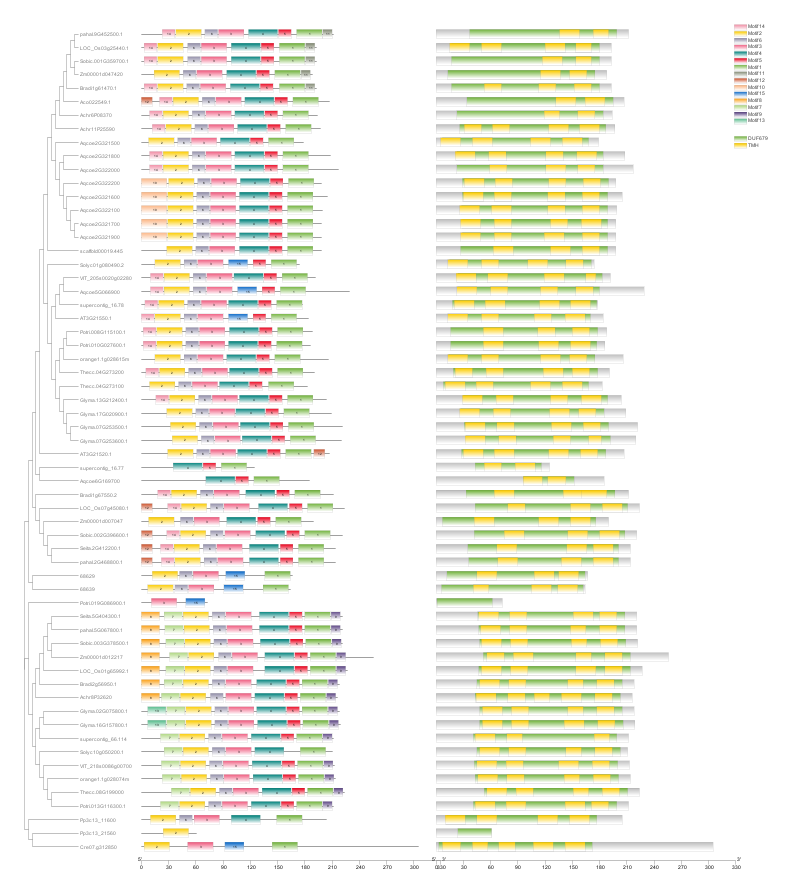

Supplement: Supplementary file 1 [file ijms-25-10435-s001.zip › Figure S2.png]

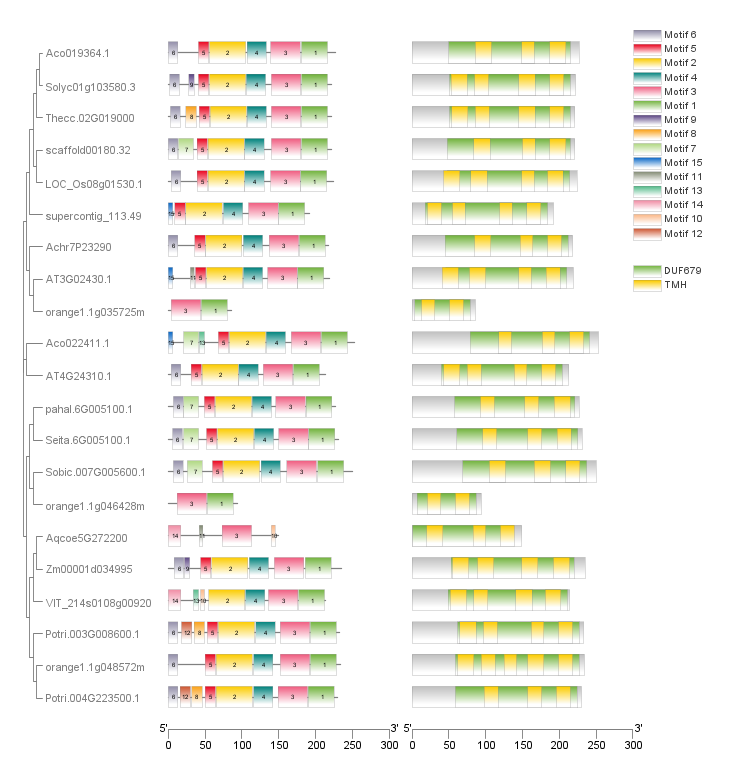

Supplement: Supplementary file 1 [file ijms-25-10435-s001.zip › Figure S3.png]

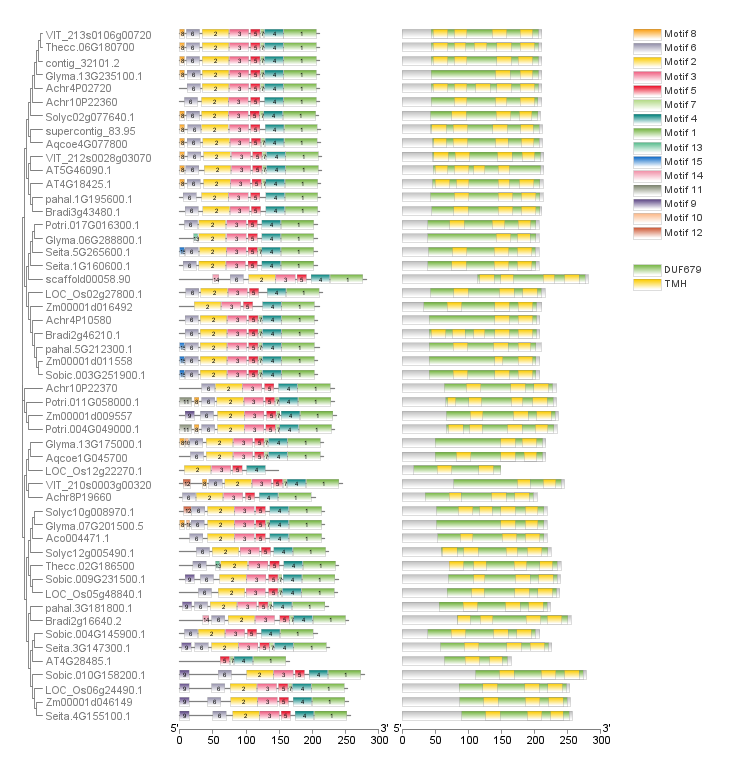

Supplement: Supplementary file 1 [file ijms-25-10435-s001.zip › Figure S4.png]

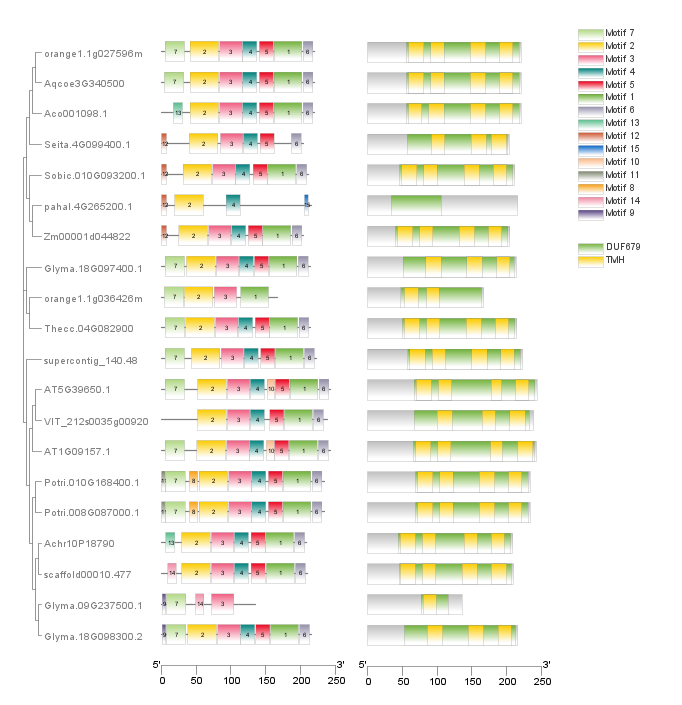

Supplement: Supplementary file 1 [file ijms-25-10435-s001.zip › Figure S5.png]

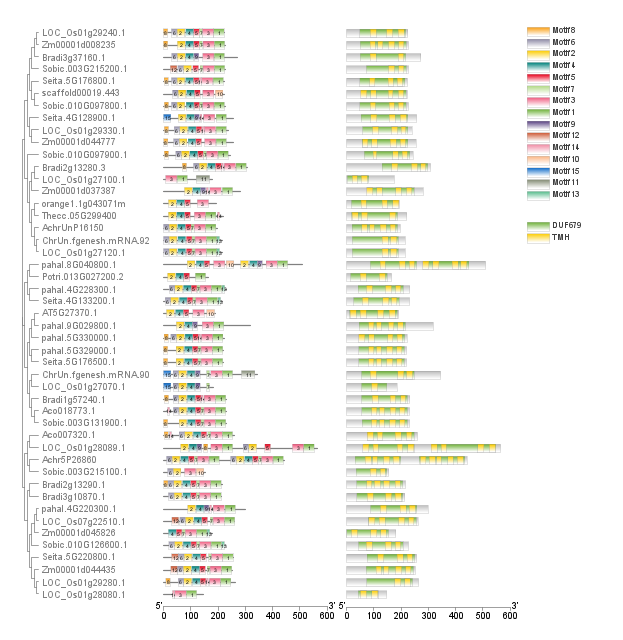

Supplement: Supplementary file 1 [file ijms-25-10435-s001.zip › Figure S6.png]
